# Carbonic Anhydrase 12 Protects Endplate Cartilage From Degeneration Regulated by IGF-1/PI3K/CREB Signaling Pathway

**DOI:** 10.3389/fcell.2020.595969

**Published:** 2020-10-16

**Authors:** Xing Zhao, Panyang Shen, Haidong Li, Yute Yang, Jiandong Guo, Shuai Chen, Yan Ma, Jiamin Sheng, Shuying Shen, Gang Liu, Xiangqian Fang

**Affiliations:** ^1^Department of Orthopaedic Surgery, Sir Run Run Shaw Hospital, Zhejiang University School of Medicine, Hangzhou, China; ^2^Key Laboratory of Musculoskeletal System Degeneration and Regeneration Translational Research of Zhejiang Province, Hangzhou, China; ^3^Department of Spine Surgery, First People’s Hospital Affiliated to the Huzhou University Medical College, Huzhou, China; ^4^The Second Clinical College, Wenzhou Medical University, Wenzhou, China

**Keywords:** endplate cartilage, carbonic anhydrase 12, IGF-1, low back pain, degeneration

## Abstract

Lumbar intervertebral disc degeneration (IVDD) is the most common cause of low back pain (LBP). Among all the factors leading to IVDD, lumbar cartilage endplate (LCE) degeneration is considered a key factor. In the present study, we investigate the effect and regulation of carbonic anhydrase 12 (CA12) in LCE, which catalyzes hydration of CO_2_ and participates in a variety of biological processes, including acid–base balance and calcification. Our results show that CA12, downregulated in degenerated LCE, could maintain anabolism and prevent calcification in the endplate. Furthermore, CA12 is regulated by the IGF-1/IGF-1R/PI3K/CREB signaling pathway. When we overexpressed CA12 in LCE, the decreased anabolism induced by inflammatory cytokine could be rescued. In contrast, reducing CA12 expression, either with siRNA, PI3Kinhibitor, or CREB inhibitor, could downregulate anabolism and cause apoptosis and then calcification in LCE. The protective effects of IGF-1 are even diminished with low-expressed CA12. Similar results are also obtained in an *ex vivo* model. Consequently, our results reveal a novel pathway, IGF-1/IGF-1R/PI3K/CREB/CA12, that takes a protective role in LCE degeneration by maintaining anabolism and preventing calcification and apoptosis. This study proposes a novel molecular target, CA12, to delay LCE degeneration.

## Introduction

Low back pain disturbs people of all ages and contributes to a large share of disease burden and disability globally (Driscoll et al., 2014; Maher et al., 2017). Lumbar intervertebral disc degeneration, according to previous literature, is the main culprit underlying LBP and spinal diseases (Golob and Wipf, 2014). Endplate cartilage is a thin layer of hyaline cartilage between the disc and vertebral bones (Chan et al., 2011), providing mechanical support by balancing the pressure between discs and nourishing the discs (Grunhagen et al., 2011). Among all the factors resulting in IVDD, endplate cartilage degeneration is considered a leading cause (Adams and Roughley, 2006). What further underscores the importance of the endplate is its direct association with low back pain (Jensen et al., 2008) and IVDD (Adams and Roughley, 2006). This is corroborated by previous studies revealing that endplate degeneration could lead to IVDD in animal models (Holm et al., 2004). Also in humans, researchers find endplate calcification responsible for IVDD by decreasing the permeability of the endplate (Rodriguez et al., 2011). Therefore, for both researchers and surgeons, endplate degeneration presents a rational and promising target for IVDD.

Carbonic anhydrase 12 (CA12) is a transmembrane protein, which includes zinc metalloenzymes that catalyze reversible CO_2_ hydration into bicarbonate and hydrogen ions (Ivanov et al., 1998). CA12 is differentially expressed in clear cell renal carcinoma (Ivanov et al., 1998), breast cancer (Barnett et al., 2008; Tafreshi et al., 2012), and colon cancer (Viikila et al., 2016). It has both a diagnostic and prognostic value in malignant diseases. In cancer cells, CA12 mainly serves as a buffer modulating the intracellular pH (Chiche et al., 2009, 2013). In cartilage, CA12 also plays an important role. It is expressed across all layers of cartilage (Schultz et al., 2011) and also functions as a pH buffer in chondrocytes (Yuan et al., 2014). In endplate chondrocytes, however, there is no research focusing on the function and mechanism of CA12.

Insulin-like growth factor 1 (IGF-1) is a hormone similar to insulin and has an anabolic effect in adults (Kraemer et al., 1991). It is reported that IGF-1 could promote extracellular matrix synthesis and cell proliferation (Steinert et al., 2008). It also impedes cartilage matrix degradation by inhibiting matrix metalloproteinases (Livneb et al., 2000). We thereby assume a potential interaction between IGF-1 and CA12 based on their common role in preventing endplate degeneration. In our study, we focus on the specific transcription factors that might regulate or enhance CA12 expression because the downstream signaling molecules of IGF-1 have been well-studied (Christopoulos et al., 2015; Ashraf et al., 2016). Moreover, interleukin-1β (IL-1β) is considered a pivotal cytokine in endplate degeneration, which increases the expression of catabolic enzymes and suppress anabolism (Chen et al., 2014; Tang et al., 2016). Other inflammatory cytokines, such as tumor necrosis factor α, also share a similar function (Neidlinger-Wilke et al., 2014). Here, we aim to clarify the cause-and-effect relation between the downregulated CA12 and those upregulated cytokines in IVDD, hoping to elucidate whether CA12 could block the effects of such cytokines on the endplate or CA12 could be regulated by these molecules.

Therefore, the present study focuses on the interaction between CA12 and endplate degeneration with further elucidation on the upstream regulation pathway of CA12 in the setting of IVDD. The purpose of our study is to explore the potential therapeutic effect of CA12 on IVDD and to provide a novel model to investigate IVDD and endplate degeneration.

## Materials and Methods

### Human Tissue Collection

Our study was approved by the institutional review board of Sir Run Run Shaw Hospital, and informed consent was obtained from each patient. Lumbar disc endplate samples in the degenerated group were obtained from patients who underwent discectomy and fusion owing to degenerative intervertebral disc disease. Lumbar disc endplate samples in the control group were obtained from patients who underwent discectomy and fusion due to vertebral burst fractures without a history of IVDD. General conditions of patients are shown in Supplementary Table S1.

### Isolation and Treatment of HEPCC and SW1353

We isolated the endplate from the lumbar discs of patients who underwent lumbar discectomy and finely diced the discs into small pieces (less than 1 mm^3^ in size) using a dissecting microscope followed by treating with 0.2% type II collagenase (Sigma-Aldrich, United States) for 1 h at 37°C. The supernatant was centrifuged, and the deposit was HEPCC. HEPCC and SW1353 chondrosarcoma cell line were cultured in Dulbecco’s modified eagle media (DMEM) supplemented with 10% FBS. An incubator was used to maintain the cells, and it was set to 37°C with 5% CO_2_ and 100% humidity. As a chondrocyte-originated cell line, SW1353 was commonly used for studying cartilage-related diseases (Radwan et al., 2015; Lu et al., 2017; Kim et al., 2020).

### HEPCC Calcification Induction

HEPCC were cultured in osteogenic induction medium for 14 days. In brief, cells were incubated at 37°C in a 5% CO_2_ and 100% humidity atmosphere. After 3 days of cultivation, non-adherent cells were removed, and the medium was replaced every 3 days. The osteogenic induction medium was made up of DMEM-HG (Hyclone, United States) supplemented with 10% FBS, which contains 0.1 μM dexamethasone (Sigma-Aldrich, United States), 10 mM β-glycerolphosphate (Sigma-Aldrich, United States), and 0.25 mM ascorbate (Sigma-Aldrich, United States).

### Transfection

The siRNA specifically targeting CA12 was constructed by RiboBIO (Guangzhou, China), and Lipofectamine RNAiMAX (Thermo Fisher Scientific) was used for siRNA according to the manufacturer’s instructions. SiRNA sequences of CA12 are shown in Supplementary Table S3.

### Virus Infection

The overexpression plasmid of CA12 was designed and constructed by Genechem (Shanghai, China). Packaging plasmids and viral vectors were cotransfected into HEK-293T cells using Lipofectamine 3000 transfection reagent (Thermo Fisher Scientific) according to the manufacturer’s instructions. Forty-eight hours after transfection, culture medium was provided for HEPCC after being centrifuged at 3,000 rpm for 10 min and supplemented with 10 μg/ml polybrene (SolarBio). Thirty-six hours after infection, 2 μg/ml puromycin was added in culture medium for selection.

### Luciferase Reporter Assay

The luciferase reporter plasmids (the full length of the CA12 promoter sequence or the mutant versions were inserted into *Xba*I restriction sites of pGL3-Firefly_Luciferase-Renila_Luciferase) were constructed by Genechem (Shanghai, China). HEK-293T cells were seeded into 24-well plates and transfected with plasmids using Lipofectamine 3000 transfection reagent (Thermo Fisher Scientific) according to the manufacturer’s instructions. The luciferase activity was measured using a dual luciferase reporter assay system (Promega, Madison, WI) 48 h after the transfection.

### Western Blotting Analysis

Cells or tissues were lysed with radio immunoprecipitation assay buffer (RIPA, Beyotime, China). Bicinchoninic acid (BCA) analysis (Beyotime, China) was used to qualify the concentration of total proteins. Protein extractions were separated by 10% SDS-PAGE and transferred onto polyvinylidene fluoride (PVDF) membranes (Sigma-Aldrich, United States). The membranes were then blocked with 5% bovine serum albumin (BSA) at room temperature for 1 h, followed by incubating with primary antibodies at 4°C overnight. After washing thrice by TBST, the membranes were incubated with secondary antibodies at room temperature for 1 h and washed thrice by TBST. Finally, the signals were detected using FDbio-Femto ECL (Fudebio, Hangzhou, China) and a chemiluminescence system (Bio-Rad, United States). The images were analyzed using Image J software (NIH).

### Quantitative Real-Time PCR (RT-PCR)

Total RNA was extracted from certain cells and tissues using an Ultrapure RNA Kit (CWBIO) according to the manufacturer’s instructions. An UltraSYBR one-step RT-qPCR kit (CWBIO) was used to quantify the expression of mRNA according to the manufacturer’s instructions. Each sample is repeated three times independently. The quantities of mRNA were normalized to β-actin. Relative primers are shown in Supplementary Table S2.

### Immunofluorescence Microscopy (IF)

HEPCC and SW1353 cells were seeded into 12-well plates and received different processes. Cells were fixed in 4% paraformaldehyde for 30 min, permeabilized in 0.5% Triton X-100 for 30 min, blocked with 5% bovine serum albumin (BSA) for 1 h and incubated with primary antibodies (diluted 1:100 by BSA) at 4°C overnight. The next day, cells were washed thrice by PBS and then incubated with secondary antibodies (diluted 1:200 by BSA) for 1 h. The nuclei were stained with DAPI. All operations, starting with the incubation of secondary antibodies, were performed in the dark. A Colibri epifluorescence microscope (Carl Zeiss, Jena, Germany) was used to acquire immunofluorescence images. The images were analyzed using Image J software (NIH).

### Alcian Blue Staining

HEPCC cells were seeded into 12-well plates and received different processes. For Alcian blue staining, cells were fixed in 4% paraformaldehyde for 30 min and then an Alcian blue stain kit (Solarbio) was used according to the manufacturer’s instructions.

### Other *in vitro* Assays

We performed other *in vitro* assays as described, including intracellular pH detection by BCECF-AM probe (Beyotime Biotec, China) (Chen et al., 2009; Li et al., 2009), cell proliferation assay by CCK-8 (Yeasen, China) (Shen et al., 2020), and flow cytometry for apoptosis (BD Biosiences, United States) (Liu G. et al., 2018).

### Biochemical Staining Assays

Human endplate cartilage cells were seeded in 24-well plates with a density of 1 × 10^5^/ml. After the treatment was acquired, alkaline phosphatase (ALP) staining (CWBIO, China), Alcian blue staining (Solarbio, China), and Alizarin red (Solarbio, China) staining was performed according to the manufacturers’ instructions.

### Chromatin Immunoprecipitation (ChIP) Assay

The ChIP assay was conducted using a SimpleChIP kit (#9005; Cell Signaling Technology) according to the manufacturer’s protocol. Before the ChIP assay, we transfected designed CA12 promoter plasmids (Supplementary Figure 5A) into 293T cells with Lipofectamine 3000 Reagent (Thermo Fisher Scientific). After transfection for 48 h, 293T were fixed with 1% formaldehyde to crosslink chromatin and protein, collected to produce chromatin fragments for incubation with normal rabbit IgG (negative control), histone H3 (positive control), and CREB (#9197; Cell Signaling Technology), respectively. The immunoprecipitates were incubated with protein G magnetic beads and then DNA fragments were separated from antibodies and protein G magnetic beads. Finally, DNA fragments were purified and analyzed by RT-PCR.

### Isolation and Culture of Disk/Endplate Specimens

For *ex vivo* study, 8-week-old male Sprague–Dawley rats were sacrificed, and the lumbar intervertebral discs were separated, including the adjacent vertebral endplates and parts of vertebrates. Hank’s solution (Solarbia, China) with 55 mM Na-citrate (Sigma-Aldrich, United States) was used to wash specimens. DMEM supplemented with 5% fetal calf serum and 20 mM Na-citrate was used to wash discs with agitation overnight in an incubator (37°C, 5% CO_2_, 100% humidity). Specimens were cultured in 48-well plates with DMEM supplemented with 10% fetal calf serum and 25 μg/ml L-ascorbate, treated with or without 10 nM IL-1β and CA12 overexpression or control adenovirus (approximately 1^∗^10E13 vg/ml) for up to 14 days. Each group had 6 specimens.

### Histological Studies

Four percent buffered paraformaldehyde was used for tissue fixing. After 1 month of decalcification in 10% EDTA, rat disc tissues were dehydrated in graded ethanol solutions and embedded in paraffin. For each paraffin-embedded specimen, three serial sections (4 μm thick) were cut on a microtome. To observe morphology and matrix degeneration in rat disc samples, the sections were stained with hematoxylin-eosin (H&E) or safranin O-fast green. To examine CA12, collagen X and osteocalcin, a histostain SABC kit (CWBIO, Beijing, China) was used for immunohistochemistry according to the manufacturer’s instructions. Samples without treatment of IL-1β (10 ng/ml) were included as a negative control. All images were acquired using a high-quality microscope. The samples were assessed independently reviewed in parallel by two experienced pathologists.

### Statistical Analysis

Statistical analysis was performed using SPSS version 18.0 software (IBM Corporation, United States). Student’s *t-*test, Fisher’s exact test, and one-way ANOVA were used for calculating the significant difference between groups. We considered *P* < 0.05 as statistical significance and presented all data as mean ± s.d. of three independent experiments.

## Results

### CA12 Expression Is Downregulated in Degenerated Lumbar Disk Tissues

**FIGURE 1:**
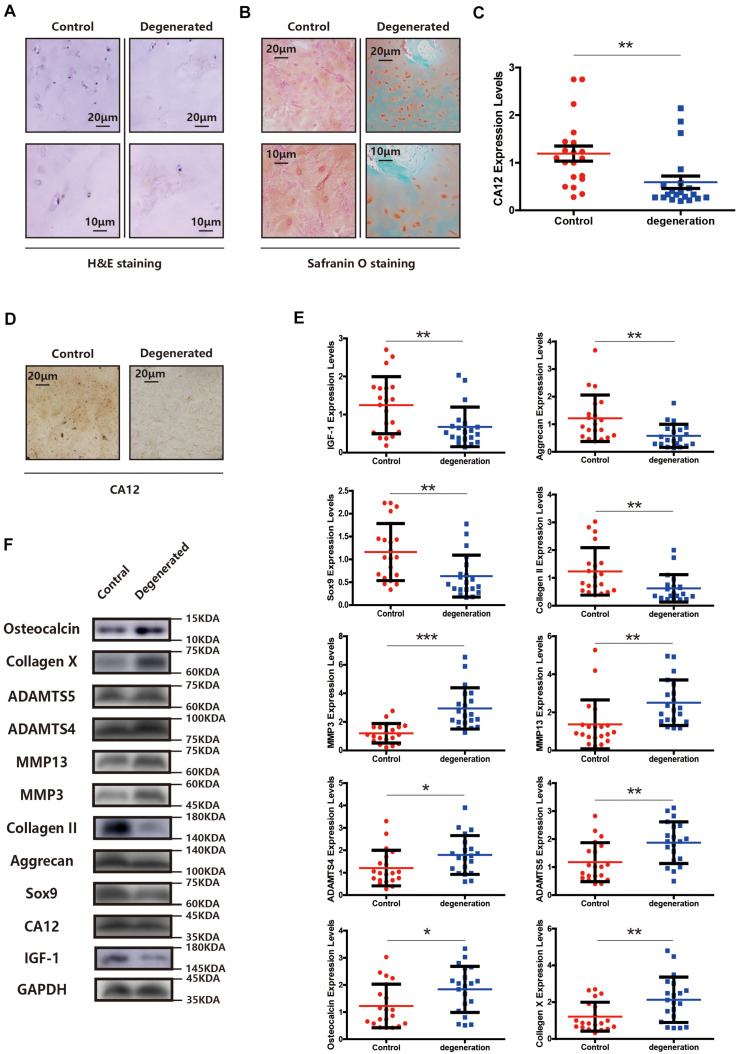
CA12 and IGF-1 expression is downregulated in degenerated lumbar disc tissues. **(A)** H&E staining in the degenerated group compared with control. **(B)** Safranin O staining in the degenerated group compared with control. **(C)** Quantitative RT-PCR of CA12 in the degenerated group compared with control. **p < 0.01. **(D)** Immunohistochemistry of CA12 between the control group and degenerated group. **(E)** Quantitative RT-PCR of IGF-1 and relative molecular in the degenerated group compared with control. *p < 0.05, **p < 0.01, ***p < 0.001. **(F)** Western blot of CA12, IGF-1, and relative enzymes in IVDD between control group and degenerated group.

We compared the demographic features, MRI findings, histopathological results [H&E staining, Safranin O/fast green staining, immunohistochemical staining (IHC), western blotting (WB), and quantitative real-time PCR (RT-PCR)] between the degenerated and control groups. There is no significant difference between two groups at the general condition aspect, presented in Supplementary Table S1. Other findings, however, show distinct patterns between degenerative and normal lumbar discs. The degenerated endplate cartilage (EPC) exhibits spindle-shaped cells with a low cell density, and the normal EPC shows homogeneous extracellular matrix interspersed with small, round, dense chondrocytes (Figure 1A). Degenerated EPC also has a decreased safranin O and more intense fast green staining compared with the control, indicating more calcification and less extracellular matrix in degenerated EPC (Figure 1B). CA12 is expressed less in degenerated EPC as evidenced by IHC, WB and RT-PCR results (Figures 1C,D and Supplementary Figures S1A, S2A). IGF-1 also is down-expressed in degenerated EPC (Figures 1E,F). In addition, in the degenerated EPC, more catabolism and less anabolism are observed (Figures 1E,F). Consistently, these results suggest a downregulation of CA12 in EPC degeneration.

### CA12 Knockdown Promotes the Degeneration and Calcification of the Lumbar Disc Endplate

**FIGURE 2:**
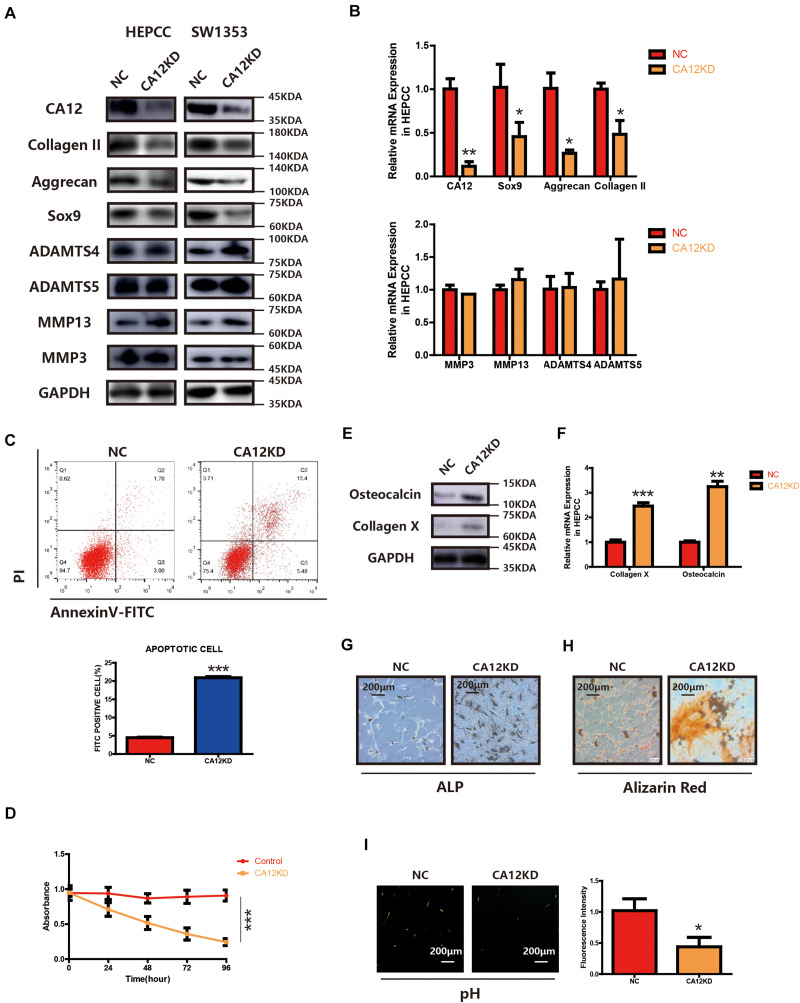
CA12 knockdown promotes the degeneration and calcification of lumbar disc endplate. **(A)** Western blot of CA12 and relative enzymes in HEPCC and SW1353 between the NC and CA12KD groups. **(B)** Quantitative RT-PCR of CA12 and relative enzymes in HEPCC between the NC and CA12KD groups. *p < 0.05, **p < 0.01. **(C)** HEPCC were transfected with siCA12, followed by annexin V-FITC/PI staining. The percentage of apoptotic cells is shown as the mean ± S.D. from the three independent experiments. ***P < 0.001, significantly different compared with the NC group. **D** SiRNA-mediated CA12 knockdown suppresses HEPCC cell proliferation as determined in the CCK-8 assay. Data represents the mean ± SD (*n* = 4). **(E)** Western blot of collagen X and osteocalcin between the NC and CA12KD groups. **(F)** Quantitative RT-PCR of collagen X and osteocalcin between NC group and CA12KD group. ***p < 0.001, **p < 0.01. **(G)** ALP staining shows more calcification in CA12KD group compared with NC group in HEPCC. **(H)** Alizarin red staining shows more calcification in CA12KD group compared with NC group in HEPCC. **(I)** Intracellular pH was decreased when CA12 was knocked down in HEPCC.

To identify the function of CA12 in the process of intervertebral disc endplate degeneration, we suppressed its expression by using CA12 siRNA (Genepharma, China) in human lumbar endplate cartilage cells (HEPCC) and SW1353 (ATCC: HTB-94). With an efficient knockdown of CA12, the mRNA and protein expressions of Sox9, Aggrecan, and Collagen II are decreased. However, there are no significant changes in MMP3, MMP13, ADAMTS4, and ADAMTS5 (Figures 2A,B and Supplementary Figure S2B). HEPCC show a higher apoptotic rate and lower proliferation rate when CA12 is knocked down (Figures 2C,D) although no significant change was observed in SW1353 (Supplementary Figure S2C). In addition, osteocalcin and collagen X are upregulated after we inhibited CA12 expression (Figures 2E,F). Considering the function and role of osteocalcin and collagen X, ALP and alizarin red staining were performed to HEPCC, which reveal consistent results (Figures 2G,H). Alcian blue staining also shows more proteoglycan in the negative control group compared with the CA12 knocked down group (Supplementary Figure S2D). The intracellular pH is decreased in CA12 knocked down cells as well (Figure 2I).

### Effects of IL-1β on IVDD Could Be Rescued by CA12 Overexpression Partially

**FIGURE 3:**
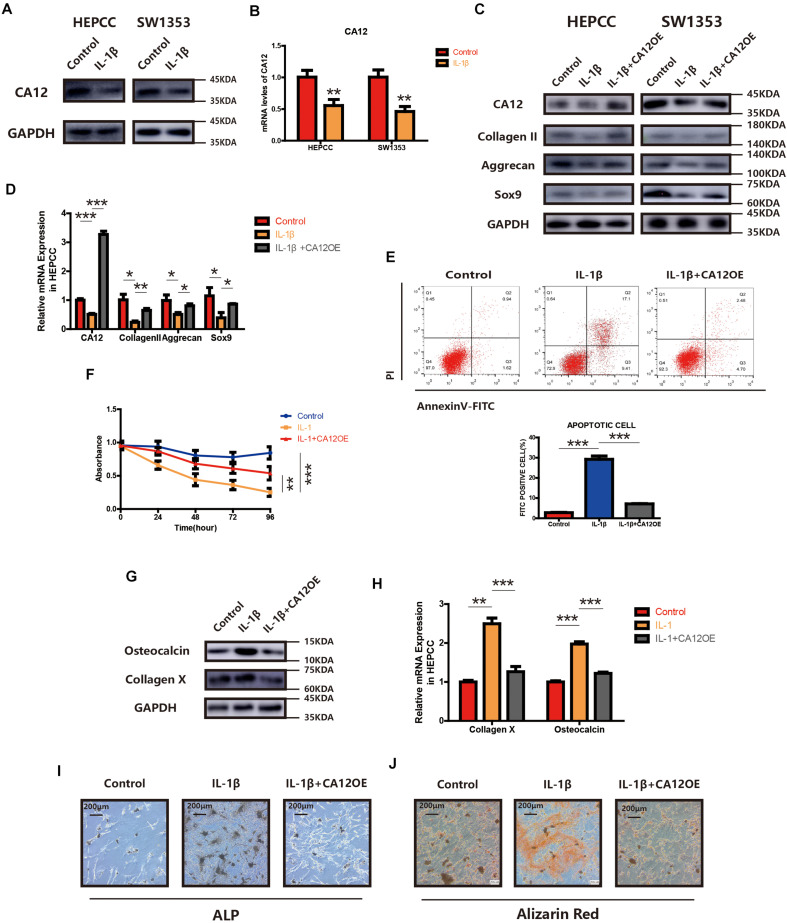
Overexpression of CA12 has an antagonistic effect on IL-1. **(A)** Western blot of CA12 in HEPCC and SW1353 treated with IL-1β (10 ng/ml). **(B)** Quantitative RT-PCR of CA12 in HEPCC and SW1353 treat with IL-1β (10 ng/ml). **p < 0.01. **(C)** Western blot of CA12, collagen II, aggrecan, and Sox9 in HEPCC and SW1353 treated with IL-1β (10 mg/ml) and the antagonism of CA12 on IL-1β. **(D)** Quantitative RT-PCR of CA12, collagen II, aggrecan, and Sox9 in HEPCC treated with IL-1β (10 mg/ml) and the antagonism of CA12 on IL-1β. *p < 0.05, **p < 0.01, ***p < 0.001. **(E)** HEPCC are treated with IL-1β (10 ng/ml) or IL-1 + CA12 overexpression plasmid, followed by annexin V-FITC/PI staining. The percentage of apoptotic cells is shown as the mean ± S.D. from the three independent experiments. ***P < 0.001. Overexpression of CA12 could antagonize the effects of IL-1 on apoptotic HEPCC. **(F)** IL-1β (10 ng/ml) suppressed HEPCC cell proliferation, and CA12 has an antagonism effect on IL-1 as determined in the CCK-8 assay. Data represents the mean ± SD (*n* = 4). **(G)** Western blot of collagen X and osteocalcin in HEPCC among the above three groups. **(H)** Real-time RT-PCR of collagen X and osteocalcin in HEPCC among the above three groups. ***p < 0.001, **p < 0.01. **(I)** ALP staining of HEPCC among the above three groups. **(J)** Alizarin red staining of HEPCC among the above three groups.

We find the expression of CA12 significantly reduced in HEPCC and SW1353 when incubated with IL-1β (Figures 3A,B). We, thus, hypothesized that the detrimental effect of IL-1β leading to IVDD may be mediated by the decrease of CA12. So we constructed a CA12 overexpressed plasmid (Genepharma, China) and transfected it into IL-1β-stimulated cell cultures, both HEPCC and SW1353. In detail, the effects of IL-1β on anabolism could be rescued by CA12 overexpression in phases of both transcription and translation (Figures 3C,D and Supplementary Figure S2E). Overexpression of CA12 could also mitigate the effects of IL-1β on cell apoptotic and proliferation in HEPCC (Figures 3E,F) but not in SW1353 (Supplementary Figure S2F). The calcification induced by IL-1β could also be reversed by CA12 overexpression (Figures 3G–J). Alcian blue staining and intracellular pH also show consistent results (Supplementary Figures S2G, S3A). IL-1β could directly induce IVDD, decrease anabolism, and promote catabolism. Taken together, CA12 is downregulated by IL-1β and CA12 overexpression could partially rescue the detrimental effects of IL-1β on IVDD.

### CA12 Expression Is Regulated by IGF-1 Through the PI3K Signaling Pathway

**FIGURE 4:**
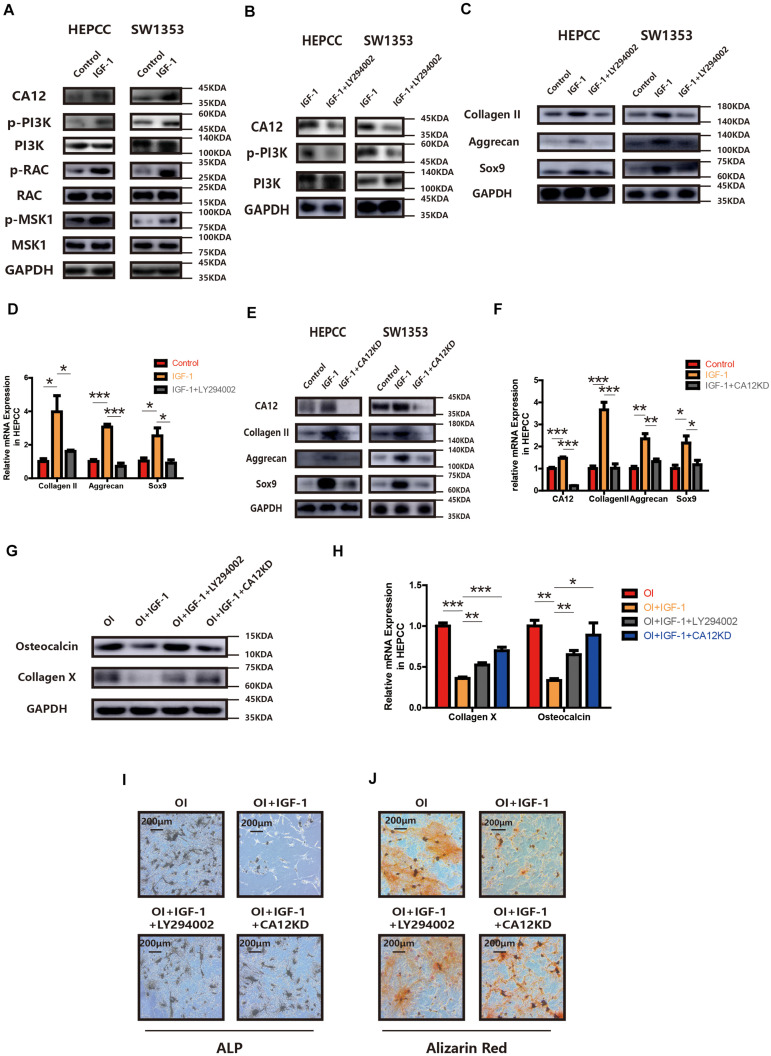
CA12 expression is regulated by IGF-1 through the PI3K signaling pathway. **(A)** Western blot analysis of CA12, RAC, p-RAC, MSK1, p-MSK1, PI3K, and p-PI3K in HEPCC and SW1353 treated with IGF-1 (100 ng/ml). **(B)** Western blot analysis of CA12, PI3K, and p-PI3K in HEPCC and SW1353 treated with IGF-1 (100 ng/ml) and LY294002 (20 μmol/l). **(C)** Western blot of Sox9, aggrecan, and collagen II in HEPCC and SW1353 treated with IGF-1 (100 ng/ml) and LY294002 (20 μmol/l). **(D)** Quantitative RT-PCR of Sox9, aggrecan, and collagen II in HEPCC treated with IGF-1 (100 ng/ml) and LY294002 (20 μmol/l). *p < 0.05, **p < 0.01, ***p < 0.001. **(E)** Western blot of CA12, Sox9, aggrecan, and collagen II in HEPCC and SW1353 treated with IGF-1 (100 ng/ml) and siCA12. **(F)** Quantitative RT-PCR of CA12, Sox9, aggrecan, and collagen II in HEPCC treated with IGF-1 (100 ng/ml) and siCA12. *p < 0.05, **p < 0.01, ***p < 0.001. **(G)** Western blot analysis of collagen X and osteocalcin in HEPCC treated with IGF-1 (100 ng/ml), IGF-1 + ly294002, and IGF-1 + CA12KD. Osteogenic inducing (OI). **(H)** Quantitative RT-PCR of collagen X and osteocalcin in HEPCC treated with IGF-1 (100 ng/ml), IGF-1 + ly294002 and IGF-1 + CA12KD. *p < 0.05, **p < 0.01, ***p < 0.001. **(I)** ALP staining of HEPCC among the above four groups. **(J)** Alizarin red staining of HEPCC among the above four groups.

Treatment with IGF-1 promotes CA12 expression with some signaling molecules (PI3K, RAC, and MSK1) phosphorylated and activated as well (Figure 4A). However, when IGF-1 is mixed with LY294002, the PI3K inhibitor, the effects of IGF-1 on CA12, its upstreaming pathway, and the promotion of anabolism are blocked (Figures 4B–D and Supplementary Figure S3B), which indicates that IGF-1 upregulates CA12 expression through the PI3K/RAC/MSK1 signaling pathway. Furthermore, when CA12 is knocked down, the effect of IGF-1 on Sox9, aggrecan, and collagen II expression is also partially blocked (Figures 4E,F and Supplementary Figure S3E), similar to the effects of LY294002. Considering the effect of CA12 on calcification, pretreatment of osteogenic inducing buffer was used in HEPCC culture. Fourteen-day-including plate ALP staining and twenty-one-day-including plate Alizarin red staining showed a protective anticalcification role of IGF-1 on HEPPC, which could be blocked by LY294002 or CA12 knock down (Figures 4I,J). RT-PCR, WB, and Alcian blue staining revealed consistent results (Figures 4G,H and Supplementary Figure S3F). Intracellular pH of HEPCC was also reduced after using LY294002 or knocking down CA12 (Supplementary Figure S3G). However, after using LY294002 or knocking down CA12, there were no significant differences of the apoptotic (Supplementary Figure S3C) and cell proliferation rates (Supplementary Figure S3D). This suggested that PI3K-inhibition or CA12 knock down has no significant effects on apoptosis or proliferation in HEPCC. These results identify CA12, upregulated by the PI3K/RAC/MSK1 pathway as an important molecule mediating the protective role IGF-1 from endplate degeneration and calcification.

### PI3K Signaling Pathway Promotes CA12 Transcription via CREB

**FIGURE 5:**
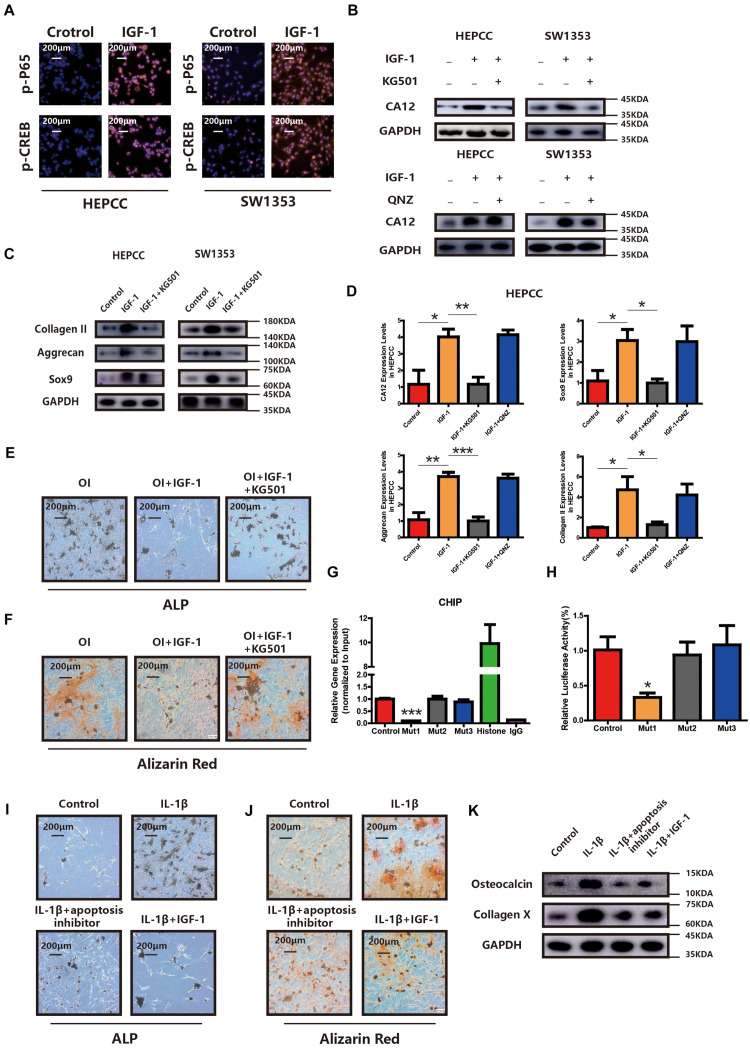
PI3K signaling pathway promotes CA12 transcription via CREB. **(A)** Immunofluorescence (IF) shows that both p-P65 and p-CREB are upregulated significantly and increased into the nucleus in HEPCC and SW1353 when treated with IGF-1 (100 ng/ml). **(B)** Western blot analysis shows that the inhibitor of CREB (KG501) blocks the effect of IGF-1 on CA12, and the inhibitor of p65 did not have this function. **(C)** Western blot analysis shows that KG501 blocks the effects of IGF-1 on collagen II, Sox9, and aggrecan. **(D)** Quantitative RT-PCR shows similar results with above Western blot analysis. *p < 0.05, **p < 0.01, ***p < 0.001. **(E)** ALP staining of HEPCC shows the calcification induced by osteogenic medium could be rescued by IGF-1 although this effect is blocked when added to KG501. **(F)** Alizarin red staining of HEPCC among the above three groups. **(G)** ChIP assay shows the specific binding site of the CREB and CA12 promoter region. **(H)** Luciferase reporter assay shows the specific binding site of the CREB and CA12 promoter region. **(I)** ALP staining of HEPCC among control, IL-1β, IL-1β + apoptosis inhibitor, and IL-1β + IGF-1 groups. **(J)** Alizarin red staining of HEPCC among the above four groups. **(K)** Western blot analysis of osteocalcin and collagen X among the above four groups.

CREB and p65 are the downstream transcription factors of the IGF-1/RAC/MSK1 signaling pathway, which is confirmed in our study by IF in HEPCC and SW1353 (Figure 5A). To determine the essential factor for CA12 transcription, inhibitors of CREB and p65 were applied, respectively. Promotion of CA12 by IGF-1 could be negated by KG501 (inhibitor of CREB) rather than QNZ (inhibitor of p65) (Figure 5B). Furthermore, promotion of anabolism by IGF-1 is blocked by KG501 rather than QNZ at both the transcription and translation levels (Figures 5C,D and Supplementary Figures S4A,D). The protective effects of IGF-1 against calcification could be reversed by KG501 (Figures 5E,F and Supplementary Figures S4G, S5H). Alcian blue staining shows a reduced proteoglycan expression after KG501 exposure (Supplementary Figure S4E). However, the apoptotic and proliferation rates show no significant change with the use of KG501 (Supplementary Figures S4C,F). Intracellular pH detected by a BCECF-AM probe shows a significantly reduced intracellular pH of HEPCC in the IGF-1 + KG501 group (Supplementary Figure S4B). To further explore the specific binding site of the CREB and CA12 promoter region, ChIP and luciferase reporter assays were performed. When the 807–818 region is mutant, the CA12 concentration and the luciferase activity are dramatically decreased, which suggests that the 807–818 region in the CA12 promoter region is the binding site for CREB (Figures 5G,H). IL-1β inducing HEPCC apoptosis could be rescued by IGF-1 or z-vad-fmk (a kind of apoptosis inhibitor) (Supplementary Figure S5B). When HEPCC is protected by IGF-1 or z-vad-fmk, IL-1β inducing spontaneous calcification in HEPCC is also prevented at the early and late stages (Figures 5I,J). Western blot analysis also provides consistent results (Figure 5K). That suggests apoptosis is an essential part of HEPCC calcification.

### The Role of CA12 in an *ex vivo* Rat Disc Model

**FIGURE 6:**
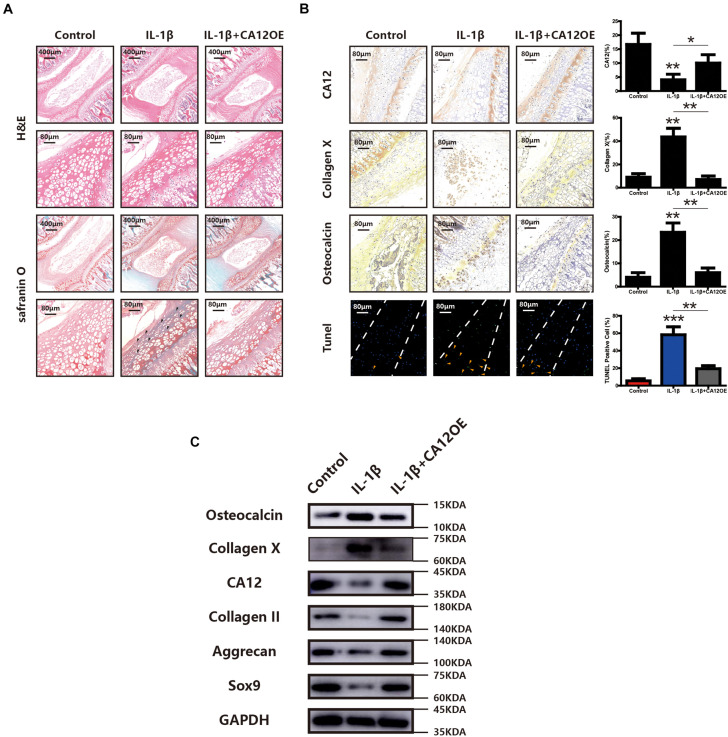
The role of CA12 in *ex vivo* rat disc model. **(A)** H&E staining and SO staining among negative control, IL-1β, and IL-1β + CA12OE groups. Degenerated area (black arrow). **(B)** IHC staining and TUNEL staining among negative control, IL-1β, and IL-1β + CA12OE groups. (TUNEL, endplate in the middle of dotted lines) *p < 0.05, **p < 0.01, ***p < 0.001. TUNEL-positive cells in endplate (yellow arrow). **(C)** Western blot for the expression of CA12, Sox9, aggrecan, collagen II, collagen X, and osteocalcin in rat intervertebral disc endplate tissue among negative control, IL-1, and IL-1β + CA12OE groups.

To further explore the potential protective effects of CA12 in degenerative disc disease, a whole disc *ex vivo* model was used in this study. According to H&E and Safranin O-fast green staining, the degeneration of ECM is more pronounced in the IL-1β group, and the degeneration could be prevented by the use of CA12 overexpression adenovirus (Figure 6A). Immunohistochemistry shows downregulated CA12 expression in the IL-1β group and upregulated expression in the IL-1β + CA12OE group. Collagen X and osteocalcin are increased in IL-1β group and decreased in the IL-1β + CA12OE group. The TUNEL assay shows that the apoptotic cells are increased in the IL-1β group and decreased in the IL-1β + CA12OE group (Figure 6B). Western blot analysis also shows the protective effect of CA12 on the endplate and even the intervertebral disk (Figure 6C). All these results indicate that CA12 could protect lumbar disc endplate from degeneration *ex vivo*.

## Discussion

Our study was initially based on a preliminary finding that CA12 is downregulated in degenerated endplates of humans. Combined with our previous data revealing an interaction between CA12 downregulation and nucleus pulposus (Chen et al., 2016), we assumed that CA12 and EPC degeneration were highly associated as well. Moreover, apart from the role of CA12 in EPC degeneration, the specific pathway regulating CA12 in EPC degeneration also remains elusive. With those questions in mind, our study finds that CA12 could protect the endplate and is regulated by the IGF-1/PI3K/RAC/MSK1/CREB signaling pathway.

Previous research has proven a protective role of IGF-1 against lumbar disc and cartilage endplate degeneration (Kim et al., 2010; Liu et al., 2015; Zhang et al., 2017). IGF-1 could also promote anabolism in extracellular matrix biosynthesis in both cartilage and lumbar discs (Martel-Pelletier et al., 1998; Kim et al., 2010), suggesting IGF-1 has protective effects. We assumed that IGF-1 might be the upstream regulator of CA12, which is validated in our study. In addition, we find that IGF-1 could activate and phosphorylate PI3K, RAC, MSK1, and CREB in HEPCC. Because the IGF-1/PI3K/RAC/MSK1/CREB signaling pathway is well established (Wiggin et al., 2002; Stitt et al., 2004; Yuzugullu et al., 2015; Dillon et al., 2015; Zhang et al., 2015; Lien et al., 2017; Liu T.J. et al., 2018), we reveal an inevitable role of this pathway in promoting CA12 expression with PI3K and CREB inhibitors. Another downstream element of the IGF-1/PI3K/RAC/MSK1 pathway, p65 (Vermeulen et al., 2003; Kefaloyianni et al., 2006) is excluded in its role of CA12 regulation by our study with p65 inhibitor QNZ. We further identify the binding site of the CREB and CA12 promoter region, which is the 807–818 region in the CA12 promoter.

As the intervertebral disk is the largest avascular organ in human, the endplate takes an important role in the transport of metabolites and nutrients (Bibby et al., 2005). It is universally acknowledged that malnutrition of IVD plays a critical role in IVDD (Bibby et al., 2005). Endplate calcification could block metabolite transportation and exacerbate IVDD by decreasing endplate permeability (Roberts et al., 1996; Wang et al., 2011). Previous research suggests that intracellular acidification is associated with calcification (Wang et al., 2010a, b), and CA12 acts by modulating intracellular pH (Chiche et al., 2009; Feldshtein et al., 2010; Doyen et al., 2012). We assume that CA12 acidifies the endplate chondrocytes and aggravates the calcification. We find that when CA12 is downregulated by IL-1β or specific siRNA, the intracellular pH decreases and HEPCC are prone to calcification. When HEPCC is induced to calcification, CA12 overexpression could prevent induction.

IL-1β has been proven as the trigger of endplate degeneration (Tang et al., 2016) and is found to be highly expressed in modic changes, a kind of endplate degeneration (Shan et al., 2017). IL-1β could promote catabolism by inducing matrix metalloproteinase expression, inhibit anabolism by suppressing extracellular matrix expression, and increase the apoptotic rate of endplate cartilage cells (Xue et al., 2015; Wang et al., 2018; Zuo et al., 2019). Endplate cartilage cell apoptosis caused by CA12 knocked down have been proven as an essential process in endplate cartilage calcification, which is similar to vascular calcification (Proudfoot et al., 2000). We validated this phenomenon and further find that CA12 overexpression could rescue the endplate chondrocytes from IL-1β stimulated apoptosis, both *in vitro* and *ex vivo*, and inhibited anabolism. In addition, CA12 is downregulated by IL-1β in HEPCC.

Taken together, we reveal that CA12 could prevent apoptosis and calcification in endplate degeneration and promote anabolism, cellular proliferation, and extracellular matrix synthesis as well. We also identify a pivotal upstream regulator of CA12, the IGF-1/PI3K/MSK1/CREB signaling pathway. Given the broad and profound role of CA12 in endplate degeneration, our study provides a promising therapeutic target for LCE degeneration and potentially IVDD. Furthermore, identification of its upstream pathway offers a deeper insight into the CA12 regulation and endows us with more potential targets for future drug development in related diseases.

However, more details are still required. First, as pointed out by previous researches, CA12 are also regulated by HIF-1 induced by hypoxia (Chiche et al., 2009; Chen et al., 2016), whether HIF-1 are regulated by IGF-1 in endplate chondrocytes remains unknown. If HIF-1 are inhibited, it remains elusive whether IGF-1 still affect CA12 expression although some papers discover the relationship in other cells and diseases (Sutton et al., 2007; Mancini et al., 2014). Second, because much previous research considers an endplate with injury as control (Tang et al., 2016; Xu et al., 2016), we follow their experience. Confounding factors, such as posttraumatic inflammation, still exist in this situation, and a normal human endplate sample would be more suitable. Third, how CA12 protects endplate chondrocytes is still unknown because we did not find ways to modulate intracellular pH accurately. Fourth, in the *ex vivo* study, CA12 is overexpressed in the whole disc, considering the close connection between nucleus pulposus, endplate, and annulus fibrosus, the therapeutic effect may not only be caused by the overexpression of CA12 in the endplate.

In conclusion, we unveil that IGF-1/PI3K/CREB/CA12 is a novel pathway in the protection of endplate degeneration and IVDD, mainly by balancing pH, modulating ECM anabolism, cell apoptosis, and cell proliferation. Therefore, genetic or pharmacological modulation of IGF-1/PI3K/CREB/CA12 activity may be a promising therapeutic approach to degenerative disc disease and a novel model for research focusing on IVDD and endplate degeneration.

## Data Availability Statement

The raw data supporting the conclusions of this article will be made available by the authors, without undue reservation.

## Ethics Statement

The studies involving human participants were reviewed and approved by the Ethics Committee of the Sir Run Run Shaw Hospital. The patients/participants provided their written informed consent to participate in this study. The animal study was reviewed and approved by the Ethics Committee of the Sir Run Run Shaw Hospital.

## Author Contributions

XZ, PS, and HL designed and conducted the experiments, analyzed the data, and wrote the manuscript. YY and JG performed *ex vivo* studies. SC, YM, and JS collected clinical specimens. GL and SS analyzed the data and critically viewed. XF conceived the work, designed the experiments, and wrote the manuscript.

## Conflict of Interest

The authors declare that the research was conducted in the absence of any commercial or financial relationships that could be construed as a potential conflict of interest.
