# Supplementary material for: Carbonic Anhydrase 12 Protects Endplate Cartilage From Degeneration Regulated by IGF-1/PI3K/CREB Signaling Pathway
Source: Front Cell Dev Biol. 2020 Oct 16;8:595969. doi: 10.3389/fcell.2020.595969 (PMC7596245; doi:10.3389/fcell.2020.595969)
Supplement: Supplementary file 1 [file Table_1.DOCX]

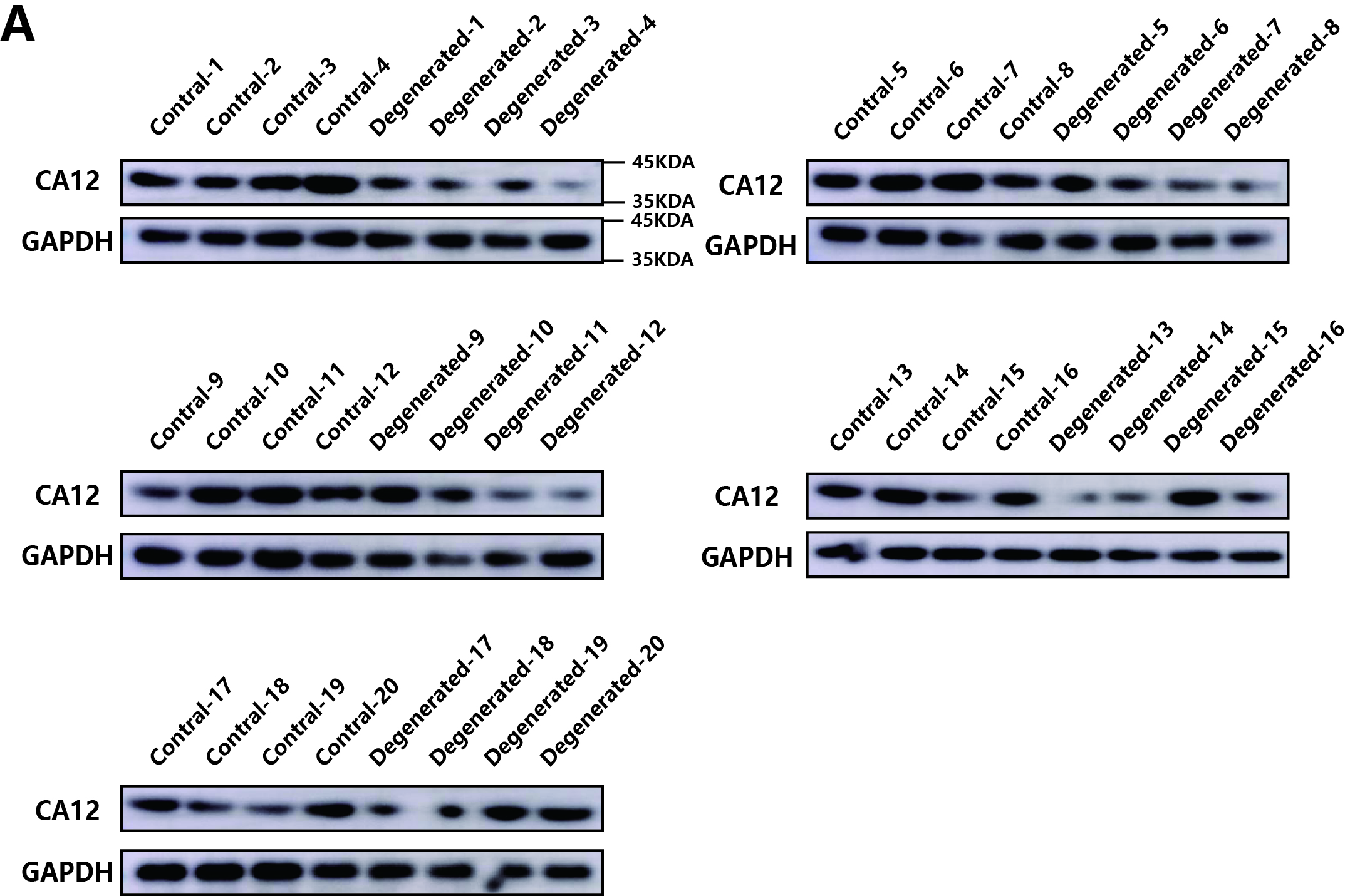


**Figure 1S A** Expression levels of CA12 in 20 degenerated samples compared with 20 control samples.


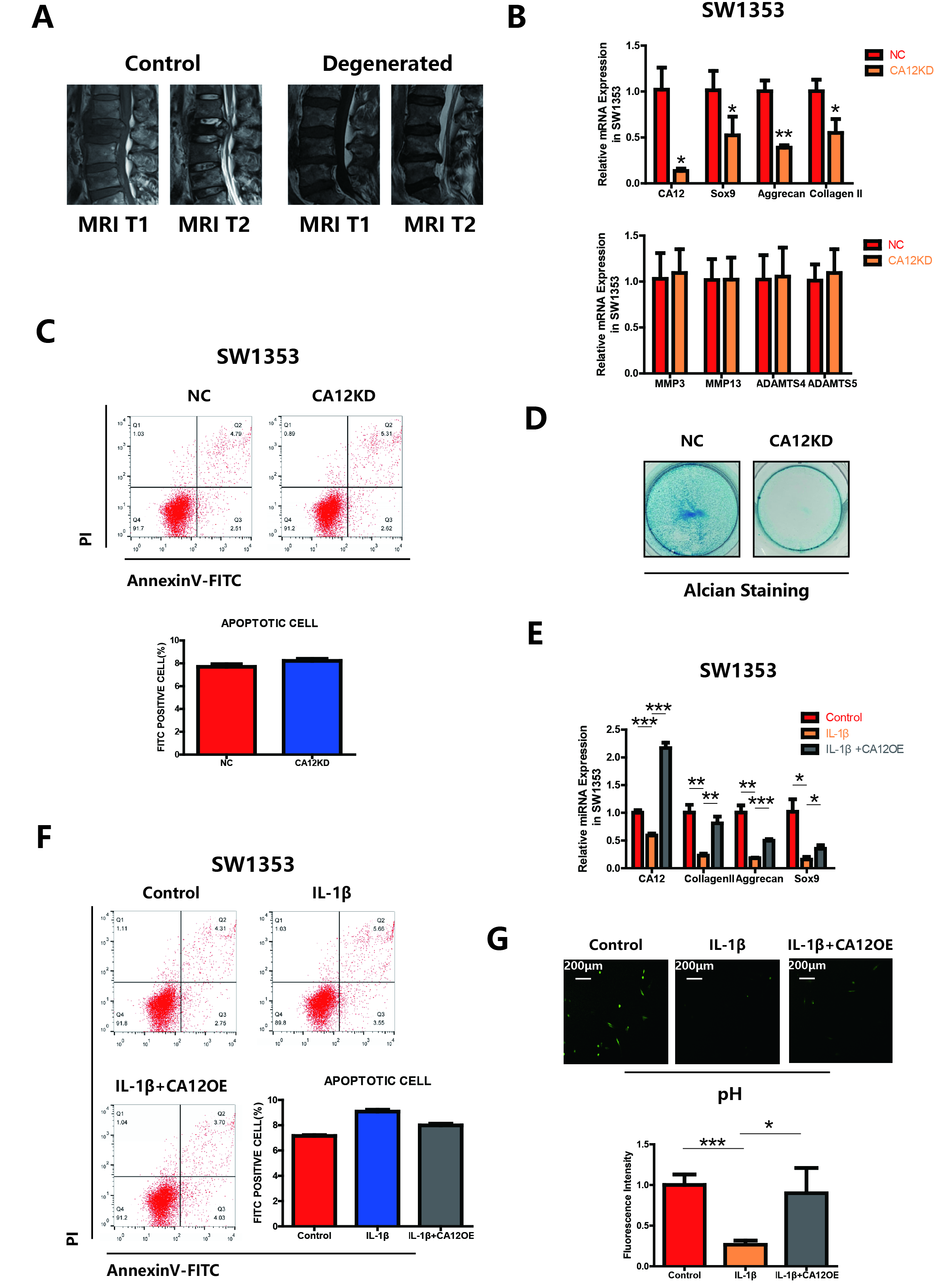


**Figure 2S A** The MRI T1 and MRI T2 of a 48-year-old female patient with L2 vertebral fracture. The intervertebral disc endplate of L1/L2 was collected in our study (Control). The MRI T1 and MRI T2 of a 55-year-old female patient with degenerative intervertebral disc disease. The intervertebral disc endplate of L4/L5 was collected in our study (Degenerated). Demographic analysis in the degenerated group compared with control. **B** Quantitative RT-PCR of CA12, IGF-1R and relative enzymes in SW1353 between NC group and CA12KD group. *p<0.05, **p<0.01, ***p<0.001. **C** SW1353 cells were transfected with siCA12, followed by Annexin V-FITC/PI staining. The percentage of apoptotic cells is shown as the mean ± S.D. from the three independent experiments. No significantly differences compared with the NC group. **D** Alcian staining of HEPCC between NC group and CA12KD group. **E** Quantitative RT-PCR of CA12, Collagen II, Aggrecan and Sox9 in SW1353 treated with IL-1β (10mg/ml) and the Antagonism of CA12 on IL-1β. *p<0.05, **p<0.01, ***p<0.001. **F** SW1353 cells were treated with IL-1β (10ng/ml) or IL-1β+CA12 overexpression plasmid, followed by Annexin V-FITC/PI staining. The percentage of apoptotic cells is shown as the mean ± S.D. from the three independent experiments. There were no significantly differences among these three groups. **G** Intracellular pH of HEPCC was significantly down-regulated in IL-1β group but this effect could be blocked by CA12.


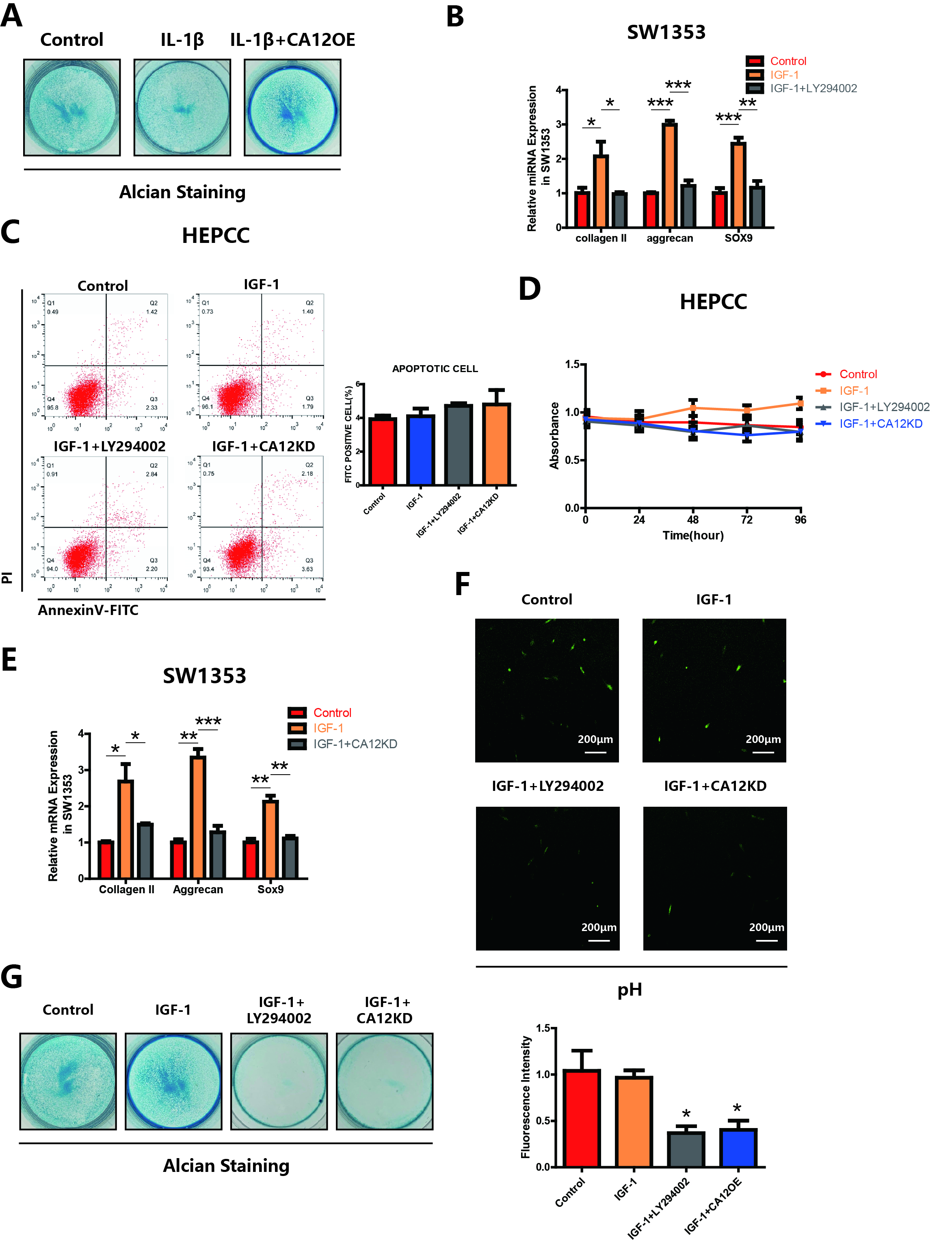


**Figure 3S A** Alcian staining of HEPCC treated with IL-1β (10ng/ml) or IL-1β+CA12 overexpression plasmid. **B** Quantitative RT-PCR of Sox9, Aggrecan, Collagen II in SW1353 treated with IGF-1(100ng/ml) and LY294002 (20µmol/l). *p<0.05, **p<0.01, ***p<0.001. **C** Cell flow assay of apoptosis showed that after treating with IGF-1 (100ng/ml), using LY294002 or knocking down CA12 in HEPCC, the percentage of apoptotic cells had no significant change. **D** CCK-8 assay showed that cell proliferation rate in HEPCC had no significantly differences among above four groups. **E** Quantitative RT-PCR of CA12, Sox9, Aggrecan, Collagen II in SW1353 treated with IGF-1 (100ng/ml) and siCA12. *p<0.05, **p<0.01, ***p<0.001. **F** Intracellular pH of HEPCC was significantly down-regulated in IGF-1+LY294002 and IGF-1+CA12KD group compared with control group and IGF-1 group. **G** Alcian staining of HEPCC among above four groups.


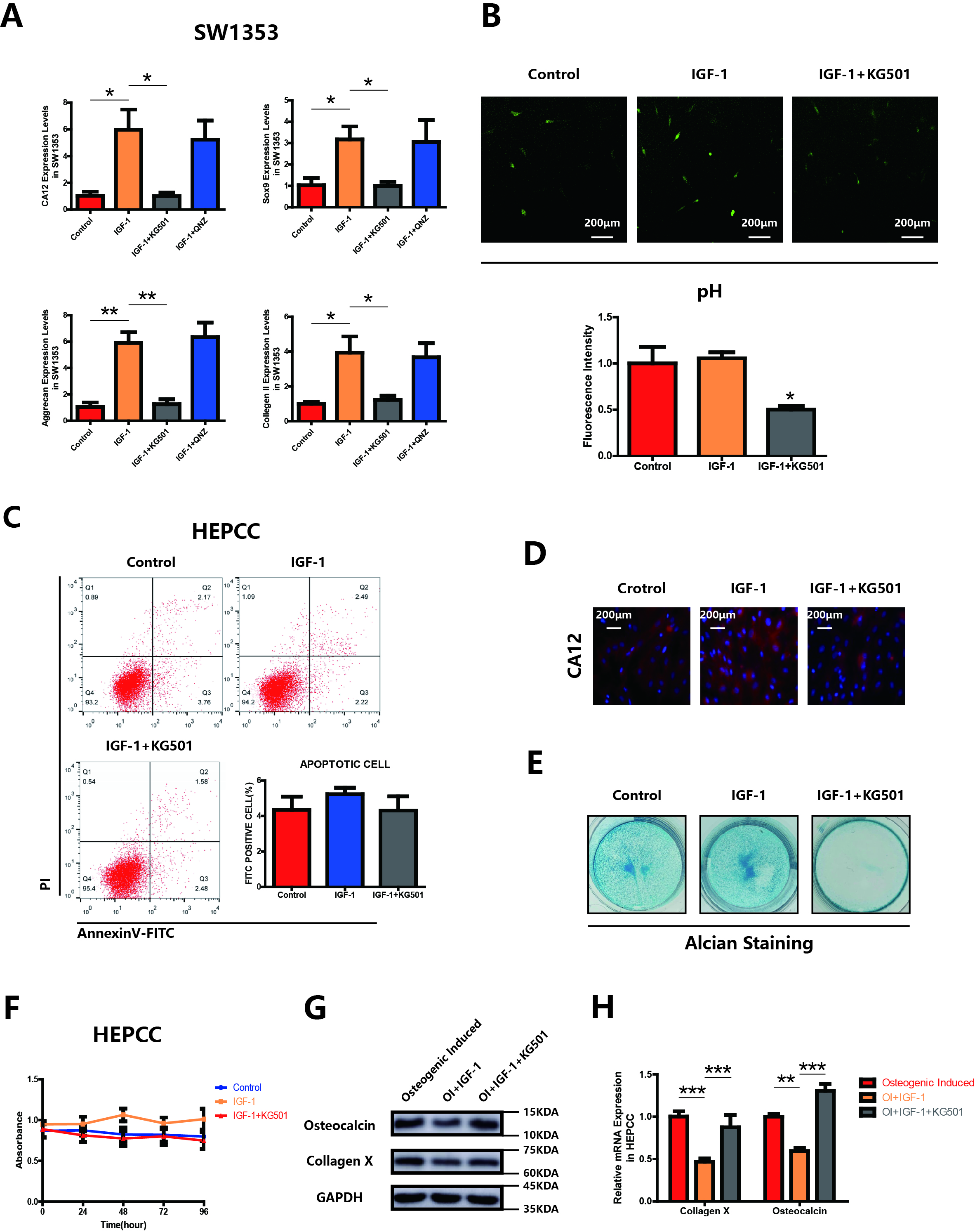


**Figure 4S A** Quantitative RT-PCR of Collagen II, Sox9, Aggrecan and CA12 in SW1353 showed that the inhibitor of CREB(KG501) blocked the effect of IGF-1 on CA12, Collagen II, Aggrecan and Sox9, while the inhibitor of p65 did not have this function. *p<0.05, **p<0.01, ***p<0.001. **B** Intracellular pH of HEPCC was significantly down-regulated in IGF-1+KG501 group compared with control group and IGF-1 group. **C** HEPCC were treated with IGF-1 (100ng/ml) or IGF-1+KG501, followed by Annexin V-FITC/PI staining. The percentage of apoptotic cells is shown as the mean ± S.D. from the three independent experiments, the percentage of apoptotic cells had no significant change. **D** Immunoflorescene (IF) showed that the effect of IGF-1 on CA12 could be blocked by KG501. **E** Alcian staining of HEPCC among above three groups. **F** CCK-8 assay showed that cell proliferation rate in HEPCC had no significantly differences among above three groups. **G** Western blot analysis of Osteocalcin and Collagen X among Osteogenic induced, OI+IGF-1 and OI+IGF-1+KG501 groups. **H** Quantitative RT-PCR of Collagen X and Osteocalcin among above three groups.


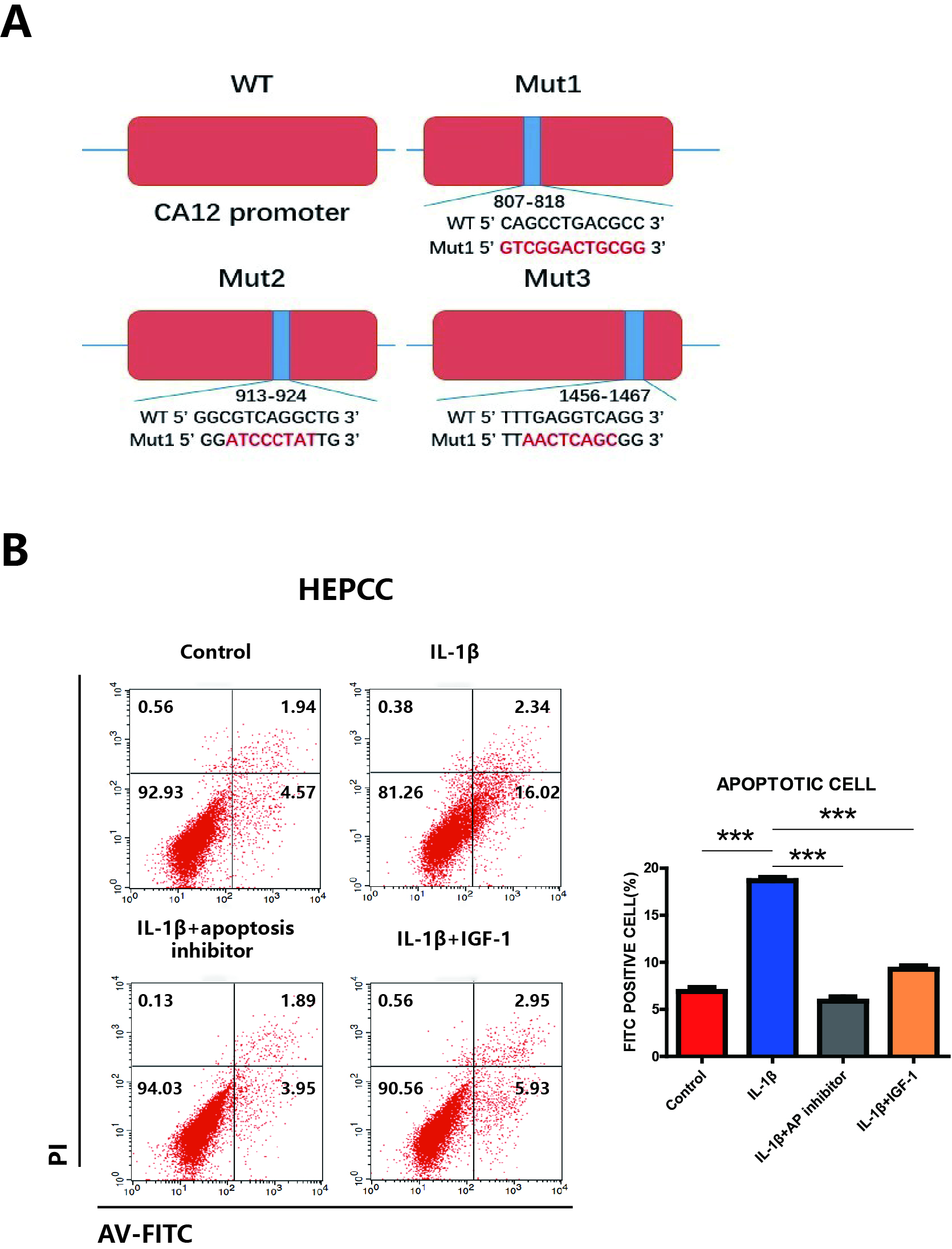


**Figure 5S A** Three predicted specific binding sites for the CREB and CA12 promoter regions. **B** Cell flow assay of apoptosis in these four groups (Control, IL-1β, IL-1β+apoptosis inhibitor, IL-1β+IGF-1).

TABLES

Table S1 GeneralConditionofPatients

| Item | Degeneration | Control | P |
| --- | --- | --- | --- |
| NumberofCases | 20 | 20 |  |
| Age(Year) | 49.5±11.4 | 45.9±10.3 | 0.853 |
| Gender(M/F) | 12/8 | 14/6 | 0.507 |
| BMI(kg/m^2^) | 22.7±1.4 | 23.3±1.7 | 0.829 |
| Operation Segment |  |  |  |
| L3-L4 | 2 | 12 |  |
| L4-L5 | 8 | 6 |  |
| L5-S1 | 10 | 2 |  |
| BMI= Weight(kg)/Height^2^ (m^2^) | | | |

There was no significant difference between two groups at the general condition aspect.

Table S2 Primer sequences used for quantitative RT-PCR

| Gene | Gene ID | Sense | Sequence |
| --- | --- | --- | --- |
| CA12  (human） | 771 | F  R | TGGCATTCTTGGCATCTGTA  TTGGTGGCTGGCTTGTAAAT |
| IGF-1  (human) | 3479 | F  R | GCTCTTCAGTTCGTGTGTGGA  GCCTCCTTAGATCACAGCTCC |
| β-actin  (human） | 60 | F  R | AGAGCTACGAGCTGCCTGAC  AGCACTGTGTTGGCGTACAG |
| MMP3  (human） | 4314 | F  R | CCTACAAGGAGGCAGGCAAG  CCCGTCACCTCCAATCCAAG |
| MMP13  (human） | 4322 | F  R | TCGGCCACTCCTTAGGTCTT  AAGTGGCTTTTGCCGGTGTA |
| ADAMTS-4  (human） | 9507 | F  R | GTCCCATGTGCAACGTCAAG  ATGCGGCCATCTTGTCATCT |
| ADAMTS-5  (human） | 11096 | F  R | GGGCACTGGCTACTATGTGG  CGTCACAGCCAGTTCTCACA |
| Sox9  (human） | 6662 | F  R | GGAATGTTTCAGCAGCCAAT  TGGTGTTCTGAGAGGCACAG |
| Aggrecan  (human） | 176 | F  R | AAGGGCGAGTGGAATGATGT  CGTTTGTAGGTGGTGGCTGTG |
| Collagen II  (human） | 1280 | F  R | CTGGAAAAGCTGGTGAAAGG  GGCCTGGATAACCTCTGTGA |
| Collagen X  (human） | 1300 | F  R | TCCCAGCACGCAGAATCCAT  GCACACCTGGTTTCCCTACA |
| Osteocalcin  (human） | 632 | F  R | TCACACTCCTCGCCCTATTG  CTCTTCACTACCTCGCTGCC |

Table S3 SiRNA sequences used for transfection

| CA12 siRNA | GTGACATCCTCCAGTATGA |
| --- | --- |
